# The Foreign Journals: Midwifery

**Published:** 1840-10

**Authors:** 


### MIDWIFERY.

*Fracture of the Upper Arm of a Child during Birth, by the natural efforts of parturition.* By Dr. LÖWENHARDT.

THE mother in this case was twenty-two years old, and it was her first labour. In consequence of slight malformation of the pelvis it was necessary to assist the delivery by the forceps; by their aid and by the moderately powerful and regular contractions of the uterus, the head had passed through the vulva (the face being turned towards the left side of the mother,) when a longer than usual pause between the pains occurred. Presently, however, they returned with considerable force, and the right shoulder, which had before been passing slowly along the perineum, was rapidly expelled; and scarcely had the left shoulder, which was directed upwards and to the right, passed through, when a snap was heard. When the trunk was completely expelled, it was found that the left arm, which lay across the chest, was fractured at the upper third of the humerus. It was put up with paste-splints and a bandage, and union took place.

The author remarks that the case is interesting, in showing that fractures of the upper extremities of new-born children are by no means always produced by awkward manipulation, but under certain circumstances are the effect of the natural process of parturition. That fractures and indentations of the skull may be produced by a projecting promontory, as well as by narrowness of the pelvis, is well known, and the author himself has met with two cases of the kind; but that fractures of the upper extremities may be affected by the act of parturition has not yet, as far as his knowledge extends, been pointed out. In this case the fracture seems to have been produced by the humerus being fixed between two hard bodies, the receding os pubis and the ribs, while the shoulder was being rapidly expelled; and consequently, a fracture of the lower extremity must be much less likely to occur, because, except in a monstrosity, the leg can never be thus impacted between two hard bodies.

*Medicinische Zeitung. Mai* 6, 1840.

---

*Successful Case of Cæsarian Section, with Suture of the Uterus.* By Dr. Godfroy, of Mayence.

On the 27th of March, 1840, Dr. Godfroy was called by M. Renant to a poor woman who had been two days in labour. The term of gestation was stated to have passed over by fifteen days. The patient was of small stature, of a slight and rickety constitution, with that malformation of the hips which gives the peculiar characteristic walk to women so affected. She was married to a man of similar constitution, and her age was forty-two. Dr. Godfroy having passed the entire hand into the vagina, discovered a portion of the cranium in the form of a wedge, the bones of which crossed each other at the sutures. The summit of the head was wedged between the sacro-vertebral angle (which was very prominent) and the pubis, this space not exceeding two inches. Delivery even with the assistance of the forceps being therefore impossible, and the woman beginning to give way under the excessive pains, it was necessary that something should be promptly undertaken. Three modes presented themselves: first to perforate the cranium and remove the trunk piecemeal; secondly, to perform symphysootomy; and, lastly, the cæsarian section. From the peculiar circumstances of the case, the first of these methods seemed fraught with great danger, and the life of the infant being ascertained was another objection. It appeared further impossible to afford a sufficient opening to give play to the forceps by dividing the symphisis, and the cæsarian section was therefore attempted. The patient was placed upon a table furnished as a bed, with her head depressed, and her legs raised and everted. Every means being adopted by emptying the bladder, &c. to render the skin lax and fix the uterus, the operator made a vertical incision from the umbilicus almost to the pubes. The integuments having been divided tissue by tissue, the peritoneum was opened cautiously, and lifted up while the incision was prolonged upwards and downwards. The folds being turned back, the uterus was carefully opened about the median line. The placenta was fixed to the anterior part of the uterus, and was readily recognized, seized, and withdrawn by the hand. The infant was very carefully removed by the feet and the umbilical cord divided; the child uttered a cry and was saved. Its weight was about six pounds and a half. No vessel required tying. The uterus was held near the abdominal orifice and cleaned, and then being left to itself contractions immediately came on, and all hemorrhage ceased. The wound in the uterus, however, did not readily unite, but left a considerable space between the edges. Some points of suture were therefore applied by means of common needles, armed with double waxed threads, and passed through the whole thickness of the wound. The reunion was perfect, and then the uterus was entirely abandoned. The abdominal orifice was closed in a similar manner through the whole thickness of the integuments, even including the peritoneum. The edges were perfectly united, except at the lower part, where a small orifice was left for any suppuration which might ensue. Charpie and a strong bandage being applied, the patient was placed in bed with her head and knees elevated.

Some slight fever arose, and the patient was twice bled. During the two succeeding days the usual sanguinolent evacuations issued from the vulva. The dressings were removed on the 1st of April, and were found scarcely soiled by suppuration. Fresh charpie, steeped in cream, was applied and left till the 6th, when the union appearing firm, the threads of the sutures were all divided and the wound simply dressed till the 24th, when there existed no trace of suppuration. There was no fever, and the patient left her bed on that day, taking care to sustain the cicatrix with a bandage. The cure was perfect.

M. Desormeaux and others have strongly objected to the sutures of the uterus. Dr. Godfroy thinks their danger has been exaggerated. At all events, the rapidity of the cure in this case without the occurrence of any accident is very remarkable.

*Gazette Médicale.* 11 *Juillet,* 1840.

---

*Case of Puerp*[illegible]*ever, with unusual Sloughing.* By Dr. LÖWENHARDT, of Prenzlau.

THE patient was a woman thirty-four years old, previously healthy and strong, who was delivered with the forceps on the 12th of November. Her five previous labours had been natural, and nothing dangerous had occurred in them, except that after the last she had had severe peritonitis.

On the 15th of November all the symptoms of peritonitis, which appeared to be especially severe in the hypogastric region, came on after exposure to cold. On the 16th and 17th she was largely bled three times, and numerous leeches were applied; purgatives and calomel and opium were also administered, but no relief was afforded. Large mercurial inunctions were then added, but the mouth was not at all affected, and the symptoms grew daily worse. On the 21st no benefit having yet resulted from the treatment, she took thirty drops of the oil of turpentine. It produced considerable pain and heat in passing urine, but on the next day there appeared to be a slight improvement in both the general and local symptoms.

On the morning of the 23d, however, though the general condition of the patient seemed to be improved, the author found a gangrenous spot as large as the palm of the hand, three inches below the umbilicus, and on cutting into it to a depth of two inches he discovered that not the slightest pain was produced. A part of the slough was removed, and pyrolignous acid, with a decoction of bark and aromatics, was ordered to be applied. Bark was also given internally with muriatic acid.

The sloughing however continued, and on the 27th it was found necessary to remove a portion of the abdominal walls, as large as the whole hand. On the day after, an opening formed into the intestine on the left side, where the sloughing extended most deeply, and a large quantity of fecal matter was discharged through it.

The patient, who had previously appeared stronger than might have been expected, now became much depressed, and as the constant fecal discharge through the wound maintained a permanent irritation, it was determined to try to close the opening into the intestine. The operation undertaken for this purpose was however unsuccessful; the intestine was found to be perforated in at least three places, and no mode of remedying its condition could be imagined. The patient became gradually more and more weak; on the 7th of December a considerable hemorrhage from the uterus occurred, and she died a few hours after.

On examination there was found at the aperture where the sloughing had passed through the abdominal walls, an excessively thickened inflamed and partly gangrenous layer of peritoneum, and beneath this the immensely swollen omentum, to which about six inches of small intestine were adherent. In this portion of intestine, which was highly inflamed, there were three apertures, of which the largest was about two inches and a half long, and was directed towards the left ilium; a second opened to the right and upwards; and a third, of an inch diameter, was situated at the lower part of the intestine. Their edges were uneven and swollen, but appeared cicatrizing.

*Casper's Wochenschrift. Mai* 16, 1840.

---

*On Phlebitis Hepatica Neonatorum.* By Dr. Schöller, Assistant Physician at the Obstetric Clinic, Berlin.

The first case related by Dr. Schöller is that of an infant, healthy for the first nineteen days of its life, when, after exposure to cold, it refused the breast, the bowels ceased to act, the abdomen became painful and tympanitic, the child vomited a greenish fluid and died within twelve hours after it showed the first signs of indisposition.

On a post-mortem examination were found a puriform effusion in the abdominal cavity, and thick layers of lymph coating the stomach, spleen, omentum, and colon. At the point where the vena portæ, and vena umbilicalis enter the liver, they were surrounded by thick layers of yellowish fibrine. On opening the vena portæ thick pus exuded, and the walls of the vessel, even in its finest ramifications, were so much thickened as to communicate to the liver considerable firmness when divided. The gall-bladder was pale, and contained a greenish mucous fluid. The spleen was pale and friable. The intestines were full of a green fluid, similar to that which the child had vomited during life.

In the second case the disease ran a still more rapid course. A female child was strong and healthy up to the twentieth day after its birth. It then refused the breast, its respiration became hurried and gasping, the abdomen hard and painful, and the child vomited a greenish fluid. The same evening trismus and convulsions came on and recurred at intervals, till, in the course of the night, the child died. An examination disclosed extensive peritonitis with effusion into the abdominal cavity, and the umbilical and portal veins were thickened and distended with pus.

On the right side was an inguinal hernia, the sac of which extended into the labium, and contained the red and swollen Fallopian tube. The unusual shortness of the round ligament of the right side seemed to have been the cause of the hernia of the tube; for the uterus had been drawn by the round ligament so forcibly to the right side as to give to its long axis an oblique direction.

*Neue Zeitschrift für Geburtskunde.* Band vii.

